# The Accumulated Health Insurance Funds

**Published:** 1921-02-19

**Authors:** 


### THE ACCUMULATED HEALTH INSURANCE FUNDS.

The Government Actuary, Sir Arthur Watson, has completed valuations of five thousand of the twelve thousand approved Societies and branches, and he has discovered a surplus of assets over liabilities amounting to £3,600,000. After allowing for certain reserves to be set aside, it is expected that a sum of between £8,000,000 and £9,000,000, for the whole of the Societies, will be available for distribution in the form of additional benefit to the members.

If such were available, we should very much like to see some statistics showing the large number of people making the weekly Health Insurance payment who never in time of sickness come upon their societies at all, and either consult a doctor who is not their panel practitioner or go to a hospital. Payment by result is a principle which does not in any precise sense belong to the administration of the National Health Insurance Acts, although it is one, as suggested by a correspondent of The Hospital in the issue of February 5, which must be attached to any perfect system of a State Medical Service.

But matters as they stand are so much the better for the panel practitioner and for those who insist, quite properly, in getting their rights from the approved societies. The latter are to have average extra benefits of £1 6s. per person. What form these benefits will take are under consideration by the societies. Some of the larger societies propose to devote a large part of their surplus to other forms of additional benefit than purely cash benefits. Dr. Addison, in a recent statement on the subject, attached great importance to contributions or benefits in aid of the maintenance and treatment of members in hospitals and convalescent homes, and he is glad to know that such provision is being actively considered.

It is felt by many that the hospitals have too long carried the National Insurance scheme on their backs; but for their aid it would long since have fallen to the ground. No doubt evidence as to this has been already given before Lord Cave's Committee of Inquiry. There was an opportunity in the amending Act of 1920, when the cash benefits were increased by 50 per cent., and the contributions were also increased at the same rate, of including hospital benefit in the scheme. As it is, the niggardly provision that arrangements may be made between a society and a hospital to secure for the institution part of the sick benefit is the only help that the law allows the hospital, and the sole means of filling the gap between what many a member gets and what he needs. The purely permissive nature of the clause is too often brought home to a hospital when it makes a suggestion that some part of the cost of maintenance of a patient should be met from National Health funds, although it must be admitted that here and there an approved society has been ready and willing to act up to what is clearly a moral obligation.

The cost of treating insured patients at the Great Northern Central Hospital has been estimated at £18,000 a year, and it is computed that 25 per cent. of the total patients come within the Act. It is suggested by this hospital that the surplus is assuredly in a measure due to the fact that a large number of young lives of insured persons were lost from war causes, and the reserves held by the societies against the future risks to these lives were accordingly released. The Societies could well afford partly to recoup the voluntary hospitals for services rendered to other insured persons during the five years to which the valuation figures refer without encroaching upon normal revenue.

How much of the present surplus will come into hospital coffers is problematical, but a strenuous effort should be made to secure against further huge accumulations largely made at the expense of our voluntary institutions.

